# Supplementary material for: A UMLS-based spell checker for natural language processing in vaccine safety
Source: BMC Med Inform Decis Mak. 2007 Feb 12;7:3. doi: 10.1186/1472-6947-7-3 (PMC1805499; doi:10.1186/1472-6947-7-3)
Supplement: Additional file 13 — RAP application directory. Sets up directory for RAP (RDF application for PHP) source code [file 1472-6947-7-3-S13.gz › rap/doc/phpdoc/__filesource/fsource_default__vocabularyVCARD_RES.php.html]

xml version="1.0" encoding="iso-8859-1"?


File Source for VCARD\_RES.php


# Source for file VCARD\_RES.php

Documentation is available at VCARD\_RES.php

```
1. <?PHP
2. // ----------------------------------------------------------------------------------
3. // vCard profile defined by RFC 2426 - Vocabulary (ResResource)
4. // ----------------------------------------------------------------------------------
5. // Version                   : 0.9
6. // Authors                   : Daniel Westphal (dawe@gmx.de)
7. //
8. // Description               : Wrapper, defining resources for all terms of the
9. //                               vCard Vocabulary. For details about vCard see: http://www.w3.org/TR/vcard-rdf .
10. //                                Using the wrapper allows you to define all aspects of
11. //                             the vocabulary in one spot, simplifing implementation and
12. //                             maintainence.
13. //
14. // ----------------------------------------------------------------------------------
15. // History:
16. // 04-04-2003                 : $VCARD_UID added. Chris Bizer (chris@bizer.de)
17. // 02-21-2003                 : Initial version
18. // ----------------------------------------------------------------------------------


24. class VCARD_RES{

26. // VCARD concepts

28. function UID()
29. {
30. return  new ResResource(VCARD_NS.'UID');

32. }

34. function ORGPROPERTIES()
35. {
36. return  new ResResource(VCARD_NS . 'ORGPROPERTIES');

38. }

40. function ADRTYPES()
41. {
42. return  new ResResource(VCARD_NS . 'ADRTYPES');

44. }

46. function NPROPERTIES()
47. {
48. return  new ResResource(VCARD_NS . 'NPROPERTIES');

50. }

52. function EMAILTYPES()
53. {
54. return  new ResResource(VCARD_NS . 'EMAILTYPES');

56. }

58. function TELTYPES()
59. {
60. return  new ResResource(VCARD_NS . 'TELTYPES');

62. }

64. function ADRPROPERTIES()
65. {
66. return  new ResResource(VCARD_NS . 'ADRPROPERTIES');

68. }

70. function TZTYPES()
71. {
72. return  new ResResource(VCARD_NS . 'TZTYPES');

74. }

76. function STREET()
77. {
78. return  new ResResource(VCARD_NS . 'Street');

80. }

82. function AGENT()
83. {
84. return  new ResResource(VCARD_NS . 'AGENT');

86. }

88. function SOURCE()
89. {
90. return  new ResResource(VCARD_NS . 'SOURCE');

92. }

94. function BDAY()
95. {
96. return  new ResResource(VCARD_NS . 'BDAY');

98. }

100. function REV()
101. {
102. return  new ResResource(VCARD_NS . 'REV');

104. }

106. function SORT_STRING()
107. {
108. return  new ResResource(VCARD_NS . 'SORT_STRING');

110. }

112. function ORGNAME()
113. {
114. return  new ResResource(VCARD_NS . 'Orgname');

116. }

118. function CATEGORIES()
119. {
120. return  new ResResource(VCARD_NS . 'CATEGORIES');

122. }

124. function N()
125. {
126. return  new ResResource(VCARD_NS . 'N');

128. }

130. function PCODE()
131. {
132. return  new ResResource(VCARD_NS . 'Pcode');

134. }

136. function PREFIX()
137. {
138. return  new ResResource(VCARD_NS . 'Prefix');

140. }

142. function PHOTO()
143. {
144. return  new ResResource(VCARD_NS . 'PHOTO');

146. }

148. function FN()
149. {
150. return  new ResResource(VCARD_NS . 'FN');

152. }

154. function SUFFIX()
155. {
156. return  new ResResource(VCARD_NS . 'Suffix');

158. }

160. function VCARD_CLASS()
161. {
162. return  new ResResource(VCARD_NS . 'CLASS');

164. }

166. function ADR()
167. {
168. return  new ResResource(VCARD_NS . 'ADR');

170. }

172. function REGION()
173. {
174. return  new ResResource(VCARD_NS . 'Region');

176. }

178. function GEO()
179. {
180. return  new ResResource(VCARD_NS . 'GEO');

182. }

184. function EXTADD()
185. {
186. return  new ResResource(VCARD_NS . 'Extadd');

188. }

190. function GROUP()
191. {
192. return  new ResResource(VCARD_NS . 'GROUP');

194. }

196. function EMAIL()
197. {
198. return  new ResResource(VCARD_NS . 'EMAIL');

200. }

202. function FAMILY()
203. {
204. return  new ResResource(VCARD_NS . 'Family');

206. }

208. function TZ()
209. {
210. return  new ResResource(VCARD_NS . 'TZ');

212. }

214. function NAME()
215. {
216. return  new ResResource(VCARD_NS . 'NAME');

218. }

220. function ORGUNIT()
221. {
222. return  new ResResource(VCARD_NS . 'Orgunit');

224. }

226. function COUNTRY()
227. {
228. return  new ResResource(VCARD_NS . 'Country');

230. }

232. function SOUND()
233. {
234. return  new ResResource(VCARD_NS . 'SOUND');

236. }

238. function TITLE()
239. {
240. return  new ResResource(VCARD_NS . 'TITLE');

242. }

244. function MAILER()
245. {
246. return  new ResResource(VCARD_NS . 'MAILER');

248. }

250. function OTHER()
251. {
252. return  new ResResource(VCARD_NS . 'Other');

254. }

256. function LOCALITY()
257. {
258. return  new ResResource(VCARD_NS . 'Locality');

260. }

262. function POBOX()
263. {
264. return  new ResResource(VCARD_NS . 'Pobox');

266. }

268. function KEY()
269. {
270. return  new ResResource(VCARD_NS . 'KEY');

272. }

274. function PRODID()
275. {
276. return  new ResResource(VCARD_NS . 'PRODID');

278. }

280. function GIVEN()
281. {
282. return  new ResResource(VCARD_NS . 'Given');

284. }

286. function LABEL()
287. {
288. return  new ResResource(VCARD_NS . 'LABEL');

290. }

292. function TEL()
293. {
294. return  new ResResource(VCARD_NS . 'TEL');

296. }

298. function NICKNAME()
299. {
300. return  new ResResource(VCARD_NS . 'NICKNAME');

302. }

304. function ROLE()
305. {
306. return  new ResResource(VCARD_NS . 'ROLE');
307. }

309. }

311. ?>
```

Documentation generated on Fri, 17 Dec 2004 16:18:19 +0100 by phpDocumentor 1.3.0RC3
